# The causal relationship between dyslexia and motion perception reconsidered

**DOI:** 10.1038/s41598-017-04471-5

**Published:** 2017-06-23

**Authors:** Sung Jun Joo, Patrick M. Donnelly, Jason D. Yeatman

**Affiliations:** 1 Institute for Learning & Brain Sciences, University of Washington, Seattle, WA 98195 USA; 2 Department of Speech and Hearing Sciences, University of Washington, Seattle, WA 98195 USA

## Abstract

It is well established that visual sensitivity to motion is correlated with reading skills. Yet, the causal relationship between motion sensitivity and reading skills has been debated for more than thirty years. One hypothesis posits that dyslexia is caused by deficits in the motion processing pathway. An alternative hypothesis explains the motion processing deficit observed in dyslexia as the consequence of a lack, or poor quality, of reading experience. Here we used an intensive reading intervention program to test the causal relationship between learning to read and motion processing in children. Our data show that, while the reading intervention enhanced reading abilities, learning to read did not affect motion sensitivity. Motion sensitivity remained stable over the course of the intervention. Furthermore, the motion sensitivity deficit did not negatively impact the learning process. Children with poor motion sensitivity showed the same improvement in reading skills as children with typical motion sensitivity. Our findings call into question the view that motion processing deficits are due to poor reading experience. We propose that the correlation between the two measures arises from other common mechanisms, or that motion processing deficits are among a collection of correlated risk factors for reading difficulties.

## Introduction

Seminal work by Lovegrove and colleagues in the 1980s reported that people with dyslexia have deficits in visual processing of transient stimuli^1^. Subsequent studies of dyslexia revealed deficits in visual sensitivity to transient and moving stimuli across a wide range of experimental conditions^2–6^. This series of studies lead to the magnocellular theory of dyslexia^5, 7^, which posits that difficulties learning to read are the consequence of a low-level deficit in the magnocellular visual pathway, and that this deficit can be detected with either physiological responses^2, 5, 6, 8^ or psychophysical thresholds^2–4, 9^ to transient or moving stimuli. However, despite the appealing simplicity of reducing the cause of dyslexia to a simple perceptual impairment, the fact that the most effective intervention programs target phonological awareness^10–12^ rather than motion sensitivity, challenges this hypothesis (but also see ref. 13). Additionally, a number of studies have failed to replicate the relationship between reading skills and motion processing using psychophysical and brain imaging measurements^14–16^.

An alternative explanation of the data relating motion sensitivity and reading skills is that motion sensitivity deficits are the consequence, rather than the cause, of dyslexia^17, 18^. Under this hypothesis, the process of learning to read changes the visual system. Deficits in motion sensitivity therefore arise from poor, or insufficient, experience with reading in people with dyslexia^17, 18^. This alternative hypothesis offers an elegant explanation for the myriad of perceptual deficits that have been reported in dyslexia, but also remains contentious since some visual processing deficits precede formal reading instruction in children with dyslexia^4, 19, 20^. Thus, to characterize the relationship between reading experience and motion processing, longitudinal studies including the relevant psychophysical measures are warranted.

Here we use an intervention study, the gold-standard for testing causal hypotheses^18^, to determine whether reading experience affects motion sensitivity. Specifically, if motion sensitivity deficits are the consequence of poor reading experience, then systematic training and practice with reading skills should lead to improved motion sensitivity^17^.

## Results

We measured motion sensitivity as indexed by estimates of motion direction discrimination threshold (percent coherence) in random dot motion stimuli (Supplementary Fig. 1; Data and code to reproduce figures available at: https://github.com/YeatmanLab/MotionDyslexia). We first assessed the relationship between reading skills and motion sensitivity from a group of children (n = 48; age = 9.48 ± 0.31) with heterogeneous reading abilities. A subset of these children with reading difficulties (n = 20; age = 9.08 ± 0.39) participated in an intensive, phonologically-based, reading intervention program (see **Methods**). To test whether motion sensitivity changes over the course of reading intervention, participants completed four psychophysical sessions, each separated by two to three weeks, in which we measured motion sensitivity (once before, two during, and once after the intervention program).

**Figure 1:**
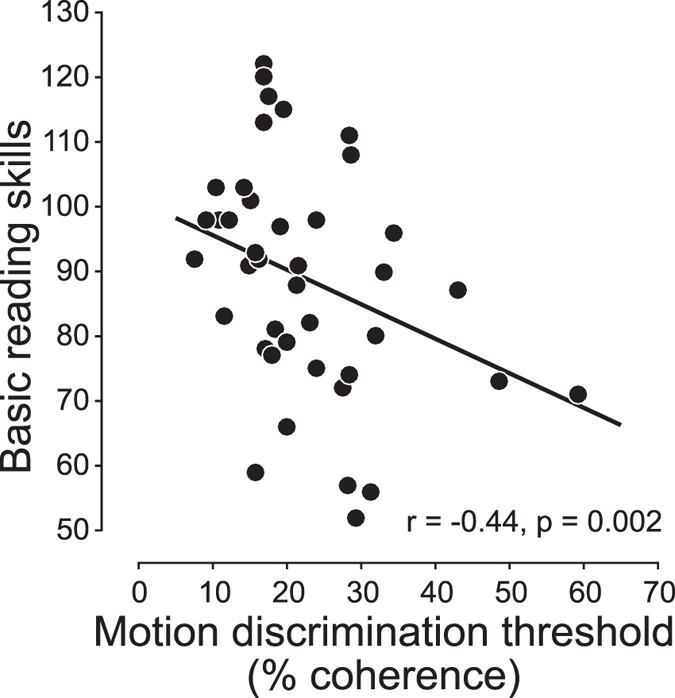
Correlation between motion sensitivity and reading skills. Basic reading skills (Woodcock-Johnson IV) are plotted as a function of motion discrimination threshold (percent coherence) for each individual.

We first established the correlation between motion sensitivity and reading skills prior to reading intervention (Fig. 1). Individuals with low motion sensitivity (high motion direction discrimination threshold) tended to have poor reading skills (Basic Reading Skills, r = −0.44, p = 0.002, 99% bootstrapped confidence interval = [−0.16, −0.65]). Furthermore, there was a group difference in motion discrimination thresholds between subjects with dyslexia (27.09 ± 3.0%) versus subjects with typical reading skills (17.89 ± 1.81%) (p = 0.02, Wilcoxon rank-sum test). Next, we asked whether reading intervention enhances motion sensitivity, and if so, how motion sensitivity is improved over the course of intervention.

**Figure 2:**
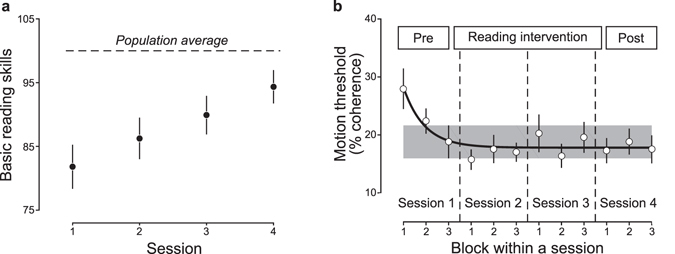
Intervention improves reading skills but not motion sensitivity. (**a**) Intervention effects. Reading skills improved significantly during the reading intervention. Reading skills are plotted as a function of time in the intervention (circle). The population average (50^th^ percentile) reading score is shown for reference. (**b**) Motion processing learning effects. Motion discrimination threshold (percent coherence) is plotted as a function of experimental block. Subjects completed three blocks during each measurement session. The black line is the best-fitting exponential decay function to the data. The shaded area is ± 1 SEM of the mean threshold in block 3 of session one (pre-intervention). Thresholds decline over the first three blocks as the subjects learn the task and then reach a stable level with no change over the intervention. Error bars represent ± 1 SEM across subjects.

The hypothesis that reading intervention enhances motion sensitivity predicts that motion direction discrimination thresholds should decrease systematically over the course of reading intervention. However, our results do not support this hypothesis. First, we found a strong, systematic improvement in reading skills across the four measurement sessions (Fig. 2a; session effects: b = 4.13, SE = 0.53, p < 10^−10^, linear mixed-effects model with subjects as a random effect). However, once motion discrimination thresholds stabilized during the first session prior to reading intervention, thresholds remained stable across all subsequent sessions as subjects underwent reading intervention. Figure 2b shows motion direction discrimination thresholds for each experimental block. The black line is the best-fitting exponential decay function to the data. Thresholds rapidly reached an asymptote within the first experimental session (before reading intervention) and then were stable across all subsequent sessions. After the first two blocks in session 1, all data points are not significantly different from the asymptote of the exponential decay function (all ps > 0.05, one sample t-test). Only the first two data points (blocks 1 and 2 in session 1) were higher than the asymptote (p = 0.02 for block 1; p = 0.07 for block 2).

Does the stability of thresholds after the first session simply reflect ceiling effects of our measurements? To rule out this possibility, we ran 32 adults on the exact same experimental paradigm. The results confirmed the dynamic range of our measurements: motion discrimination thresholds in adults were significantly lower (14.83%, bootstrapped 95% confidence interval = [12.65, 16.38]) than the asymptote (17.80%) in children. These results demonstrate that, despite a systematic improvement in reading skills, reading intervention does not enhance motion sensitivity.

A further implication of these results is that it is essential to consider task-learning, or practice effects, when making longitudinal measurements. The significant decrease in thresholds that occurred over the first three blocks of pre-intervention measurements (150 trials) is likely to reflect either the time it takes subjects to learn how to perform the task at threshold, or changes in attention, focus and lapse-rate that follow from practice. These results highlight an important methodological issue for intervention studies. If we had only run one block in each session rather than three, we would have then observed a significant increase in performance over the first three sessions, which might have been interpreted as an intervention effect rather than a practice effect.

We have shown that learning to read does not enhance motion sensitivity. Next, we considered whether motion sensitivity deficits have a negative impact on the process of learning to read. If motion processing deficits are the primary cause of dyslexia, and prevent children from learning to read, then subjects with low motion sensitivity should improve less than subjects with high motion sensitivity during the reading intervention. We tested this hypothesis by dividing our sample into two groups: low motion sensitivity vs. high motion sensitivity (median-split based on the initial motion discrimination threshold).

**Figure 3:**
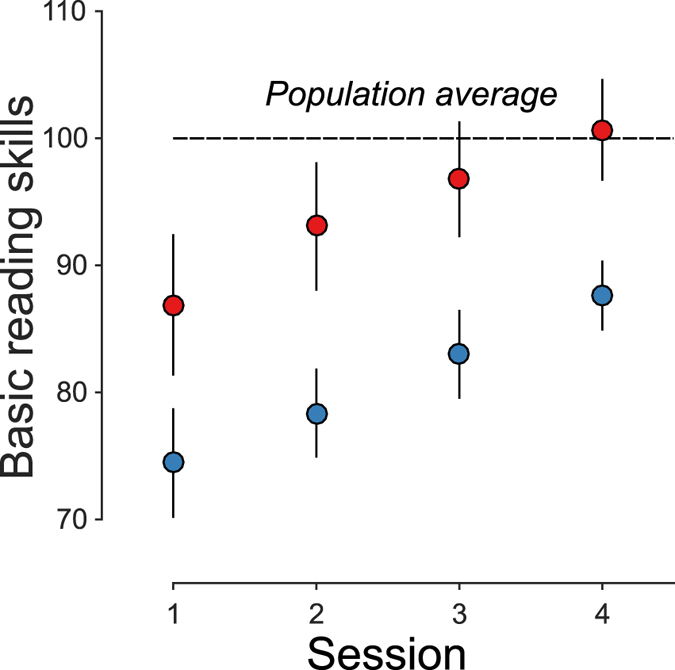
Response to intervention is equivalent for subjects with low and high motion sensitivity. Reading skills (y-axis) are plotted at each time point for the low motion sensitivity group (blue) and high motion sensitivity group (red). The error bars are 68% bootstrapped confidence intervals (Cis). Mean growth rate and bootstrapped 95% CI (CI_95_) were 4.47 (CI_95_ = 2.11 to 7.22) for high motion sensitivity group versus 4.40 (CI_95_ = 2.44 to 6.58) for the low motion sensitivity group. Both groups showed similar improvements over the intervention.

Contrary to this prediction, both groups show linear increases in reading skills with nearly identical amounts of change over the intervention (Fig. 3). Furthermore, there is no correlation between initial motion sensitivity and learning growth rate (Supplementary Fig. 3; r = 0.05, p = 0.84). Thus, our results show that, despite the initial low reading skills, subjects with low motion sensitivity still responded to reading intervention as much as subjects with high motion sensitivity, and that low motion sensitivity is not an accurate predictor of a child’s reading growth rate.

## Discussion

We tested the hypothesis that reading experience enhances motion sensitivity and provided strong evidence against this causal interpretation. An alternate causal interpretation predicts that motion sensitivity should predict children’s response to intervention. We found that subjects with low motion sensitivity, despite having initial low reading skills, showed similar learning slopes compared to subjects with high motion sensitivity (Fig. 3). Taken together, these findings support the view that motion sensitivity and dyslexia might be indirectly related through another common mechanism^21^, or that poor motion processing is just one of many factors that contribute to reading difficulties.

We found that improvements in motion sensitivity occur during the first 150 trials (three blocks) of the motion discrimination task (Fig. 2b), and that thresholds reach asymptote by the last block of the first session and remain stable over the course of reading intervention. The changes in sensitivity that occur over the first three blocks follow the typical exponential function that characterizes practice effects in perceptual learning paradigms^22^, and are likely to reflect attention and task learning, rather than changes in sensory processing. This finding has two important implications for re-interpreting the literature on motion sensitivity and reading.

First, a recent study by Olulade and colleagues used functional magnetic resonance imaging (fMRI) to assess whether reading intervention affects blood oxygen level dependent (BOLD) responses in hMT + , the motion processing hub of the brain^17^. They found that hMT + responses increased after reading intervention, but not after a control period, suggesting that reading experience might improve motion processing. However, if the hMT + response shows the same practice/task-learning effect as behavioral thresholds, then we would expect to see changes over time that do not necessarily reflect reading experience. In line with this interpretation, a majority of subjects (73%) went through the reading intervention before the control period, putting them on the steep part of the task-learning curve (Fig. 2b). However, it is also important to note that BOLD responses in hMT + reflect not only sensory processing of the stimulus^23, 24^, but also on top-down signals due to attention and cognitive demands of the task^25–27^. Therefore, it is possible that reading intervention alters other sources of signals affecting hMT + BOLD responses, without changing motion processing per se.

Second, our findings offer an explanation for why some studies have failed to replicate the motion sensitivity deficit in dyslexia^15, 16^. Reading scores changed substantially when our subjects participated in a targeted intervention program, but the motion sensitivity deficit remained relatively stable. For example, some of our intervention subjects had reading scores in the normal range after the intervention, but still had poor motion sensitivity. If a future study included these children as control subjects (based on their typical reading scores), it would bias estimates of motion sensitivity for the control group. Unfortunately, we as researchers frequently do not know subjects’ prior experience, whether they have or have not attended intervention, and if so, what type of intervention and for how many hours. Thus, it is important to consider the influence of differences in educational experience within the sample when conducting correlational studies on developmental dyslexia.

Without a clear causal relationship, why might motion sensitivity be correlated with reading skills? We hypothesize that both motion sensitivity deficits and reading disabilities arise from other common mechanisms, or that motion processing deficits are among a collection of correlated risk factors for reading difficulties. For example, motion sensitivity is typically measured using a perceptual decision-making paradigm, during which the visual system accumulates evidence for perceptual judgments^28–32^. It is possible that integrating motion information rather than motion detection is impaired in dyslexia^33^. Learning to correctly recognize sequences of letters, and associate them with their proper sound and meaning, might depend on a similar underlying evidence accumulation or decision-making mechanism. Dyslexia is a multifaceted impairment in learning to read and it is unlikely that there is a single, unique cause of dyslexia. Rather, a collection of correlated risk factors increase the likelihood that a child will struggle with learning to read^34^. In line with multiple deficit models of dyslexia^35^, a deficit in the motion processing pathway, or in the decision-making network in general, might be one of the many contributing factors—such as impaired phonological processing, auditory processing, and visual attention—to reading difficulties in developmental dyslexia.

## Methods

### Subjects

Each participant provided written informed consent under a protocol that was approved by the University of Washington Institutional Review Board and all methods were carried out in accordance with these guidelines. Based on a literature review, we expected a correlation coefficient of r = 0.40 between motion sensitivity and reading scores. We conducted a power analysis to determine the number of subjects to needed to detect the correlation between motion sensitivity and reading skills using type I error (0.05) and type II error (0.2). The number of subjects was 47. Thus, we planned to recruit ~50 subjects. A total number of 48 subjects (23 female, 25 male) participated in the study. All subjects were native English speakers with no history of neurological damage or psychiatric disorder. All subjects had normal or corrected-to-normal vision, and gave informed written consent in accordance with the University of Washington Institutional Review Board. To define the criteria for dyslexia, we used Woodcock-Johnson IV (WJ-IV) Word Identification (WID, single real word reading), and Woodcock-Johnson Word Attack (WA, single pseudo-word reading)^17^. Our criteria were the WJ-IV WID score or WA score < 90. Twenty subjects (9 female, 11 male; age = 9.08 ± 0.39) from the total subject pool participated in an intensive reading intervention program. These intervention subjects were recruited based on parent reports of dyslexia diagnosis and/or severe reading difficulties. Seventeen out of the twenty subjects met the criteria for dyslexia. However, we included all the subjects in the study so that we would have a range of motion discrimination thresholds, allowing us to examine how individual differences in motion sensitivity affect learning trajectories during the intervention. Furthermore, excluding 3 subjects who did not meet the criteria for dyslexia did not change the main pattern of results (Supplementary Fig. 2).

### Experimental sessions

Subjects in the intervention group participated in four experimental sessions that occurred at regular intervals before the intervention, after 2.5 weeks of intervention, 5 weeks of intervention and post-intervention. During each experimental session subjects completed the motion direction discrimination experiment (“The Space Race”, Supplementary Fig. 1) and a battery of behavioral tests including subtests from the Wechsler Abbreviated Scales of Intelligence, Comprehensive Test of Phonological Processing, Test of Word Reading Efficiency and the Woodcock Johnson IV Tests of Achievement. Here we report the Woodcock Johnson Basic Reading Skills composite score because it includes measures of real-word and pseudo-word reading and is the most widely used, standardized index of reading abilities.

### Reading intervention

Intervention subjects were enrolled in 8 weeks of the *Seeing Stars: Symbol Imagery for Fluency, Orthography, Sight Words, and Spelling* ^36^ at three different *Lindamood-Bell Learning Centers* in the Seattle area. The curriculum consists of directed, one-on-one training in phonological and orthographic processing skills. This curriculum utilizes an incremental approach, building from letters and syllables to words and connected texts, and employs a multi-sensory approach to systematically train the building blocks of skilled reading. For example, to develop a robust understanding of letters, and the sounds they represent, children are taught to attend to their mouth movements as they produce letter sounds, practice air-writing the shape of letters as they visualize the symbol associated with the sound and then practice combining symbols and sounds to make new pseudo- and real-words. Each lesson is built around a series of tasks that encourages students to visualize the letters that they see and the sounds that they hear and associate the multisensory components of printed text. Utilizing imagery of orthography, phonology and meaning, *Seeing Stars* builds systematically from phonology, to decoding and spelling skills, to comprehension. This intervention program has been described in more detail in other publications^17^.

### Stimuli and apparatus

Stimuli were created using MATLAB (The Mathworks Corporation, Natick, MA, USA) in conjunction with the Psychophysics Toolbox^37, 38^ on a Linux PC (Mint Mate, version 17). Stimuli were displayed on a LG liquid crystal display (1,920 × 1,080 resolution, 120 Hz refresh rate, subtending 51° horizontally). The subjects’ response was collected using a joystick. The viewing distance was 56 cm. We used random-dot motion stimuli (150 dots) that were displayed in a circular aperture (14° in diameter) centered around the fixation mark (1°) at the center of display. Light (271 cd/m^2^) and dark (0 cd/m^2^) dots (dot size = 0.15°) moved at the speed of 8°/s on a gray background (135 cd/m^2^). Each dot was assigned a random life-time from a uniform distribution between 0 and 200 ms (24 video frames). When a dot’s lifetime expired, it was randomly re-positioned within the aperture and assigned the maximum lifetime (200 ms). Motion coherence was defined as the percentage of dots moving together in the same direction compared to dots moving in random directions.

### Procedure

Each session (on a day) comprised 3 experimental blocks with the exception of some control subjects who only completed 2 experimental blocks. At the beginning of each session, subjects completed 10 practice trials comprising high coherence motion (60–100%). In each experimental block, there were two independent QUEST^39^ staircases to measure motion direction discrimination threshold. Each staircase had 20 trials and 10 extra trials were randomly inserted during each experimental block in which 70% coherence motion was displayed (50 total trials per block). These easy trials were used to engage subjects in the task and monitor their lapse rate.

Each trial started with a fixation mark at the center of the display. After 500 ms, random-dot motion stimuli were displayed for 600 ms. Subjects used a joystick to report the motion direction. The fixation mark was turned off when the response was made, and visual and auditory feedback was given for to indicate correct and incorrect responses. The experiment did not proceed until subjects reported the motion direction. The inter-trial interval was 1 s, and after this interval the fixation mark re-appeared at the center of the display to indicate the beginning of the next trial.

### Data analysis

The overall performance on the easy trials were above 90% in each session (mean ± SEM: session 1, 90.56 ± 2.9%; session 2, 96.11 ± 2.0%; session 3, 95.56 ± 1.5%; session 4 93.33 ± 1.8%), confirming that our subjects were engaged in the task. We excluded from the main analysis all experimental blocks in which the lapse rate on these easy trials were above 30% (16 out of 432 blocks).

We re-fitted the data from the staircases to a Weibull function with both threshold and slope as free parameters to estimate the threshold. If the resulting threshold estimate was greater than 100%, we discarded that staircase. The remaining threshold estimates from the experimental block was averaged.

To assess the subjects’ learning function, we fitted these thresholds estimates from the 12 experimental blocks to the following exponential decay function:

$${\rm{y}}={\rm{a}}+{\rm{b}}\ast \exp (-x/t),$$

where b is the initial threshold estimate averaged across subjects, a is the asymptote, and t is the time constant. Both a and t are free parameters.

To assess the growth rate of reading skills in children with low (n = 8) versus high motion sensitivity (n = 9), we split our intervention subjects into two groups based on the median value (20%) of the motion discrimination thresholds in the first session. Three subjects were excluded for this analysis because their threshold estimates for the first session could not be reliably measured.

### Data availability

All code and data associated with this manuscript is available at: https://github.com/YeatmanLab/MotionDyslexia.

## Electronic supplementary material

SUPPLEMENTARY INFO
