# Supplementary material for: The causal relationship between dyslexia and motion perception reconsidered
Source: Sci Rep. 2017 Jun 23;7:4185. doi: 10.1038/s41598-017-04471-5 (PMC5482857; doi:10.1038/s41598-017-04471-5)
Supplement: Supplementary file 1 — SUPPLEMENTARY INFO [file 41598_2017_4471_MOESM1_ESM.pdf]

Supplementary Information

The causal relationship between dyslexia and motion perception reconsidered

Sung Jun Joo[1,2], Patrick M. Donnelly[1,2], & Jason D. Yeatman[1,2,*]
[1]Institute for Learning & Brain Sciences, [2]Department of Speech and Hearing Sciences,
University of Washington, Seattle, WA 98105, USA

a

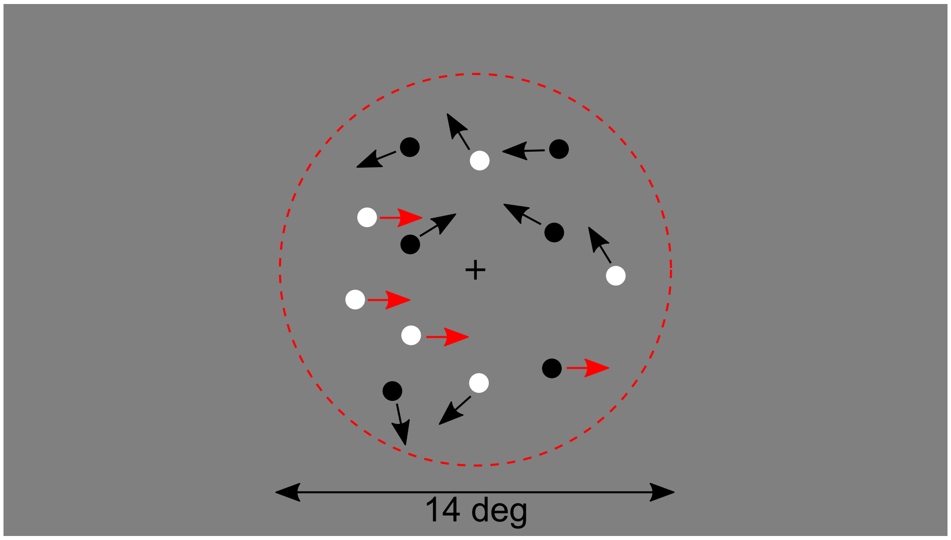

Supplementary Figure 1. A schematic of random dot motion stimuli. a, Light and dark dots were displayed in a circular aperture (indicated by the red dashed line which was not shown in the experiment) at the center of the display. Motion coherence was defined as the percentage of dots moving in the same direction (red arrows) among all dots. Noise dots (black arrows) moved in a random direction. During the practice session, participants practiced motion direction discrimination in the context of a game called "The space race".

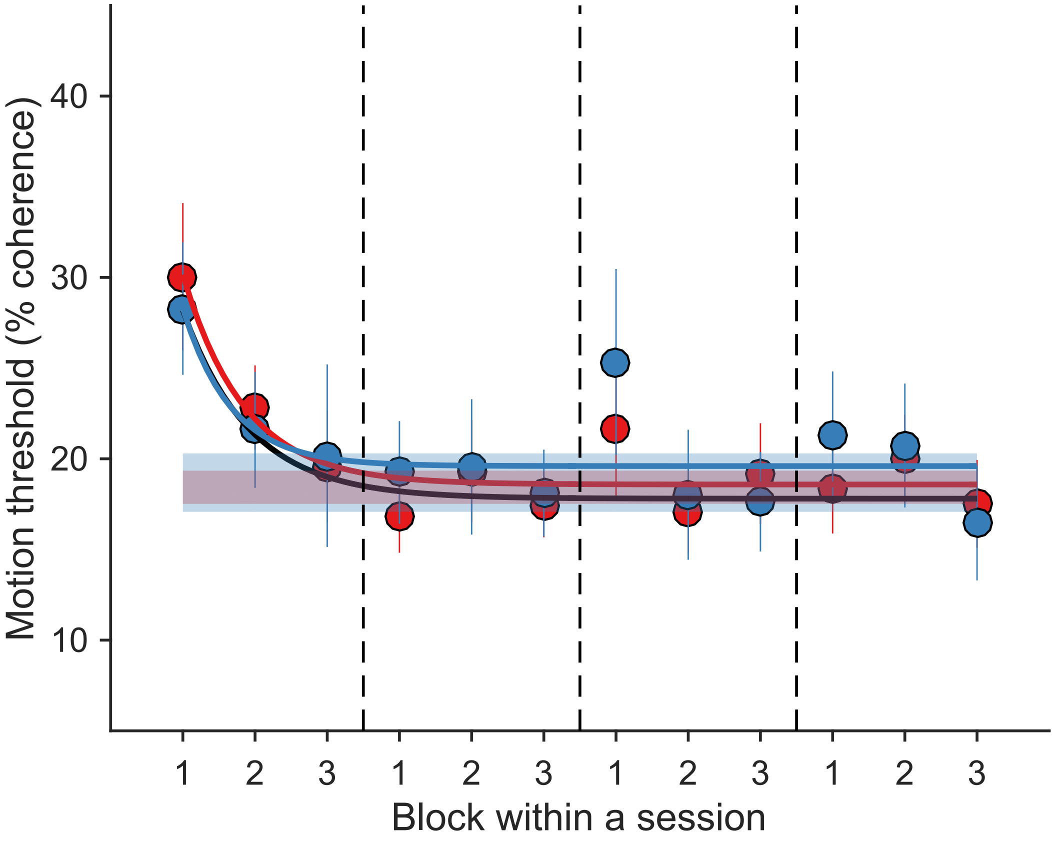

Supplementary Figure 2. Motion learning effects are not affected by different criteria for defining dyslexia. We reanalyzed data by applying two different criteria to define dyslexia. Red and blue data points are motion thresholds across the intervention period for different inclusion criteria: WJ-IV WID score or WA score < 90 (red, n = 17) and WJ-IV WID score or WA score < 85 (blue, n = 11), respectively. Each colored line is the best-fitting exponential decay to the data. Color-shaded areas represent 68% CIs of the bootstrapped distribution (5000 resampled) of the asymptote for each inclusion criterion. The black line is the result in the main text (Fig. 2b). Applying different criteria for dyslexia did not affect our results: all curves show stable motion thresholds across the intervention period after the first session.

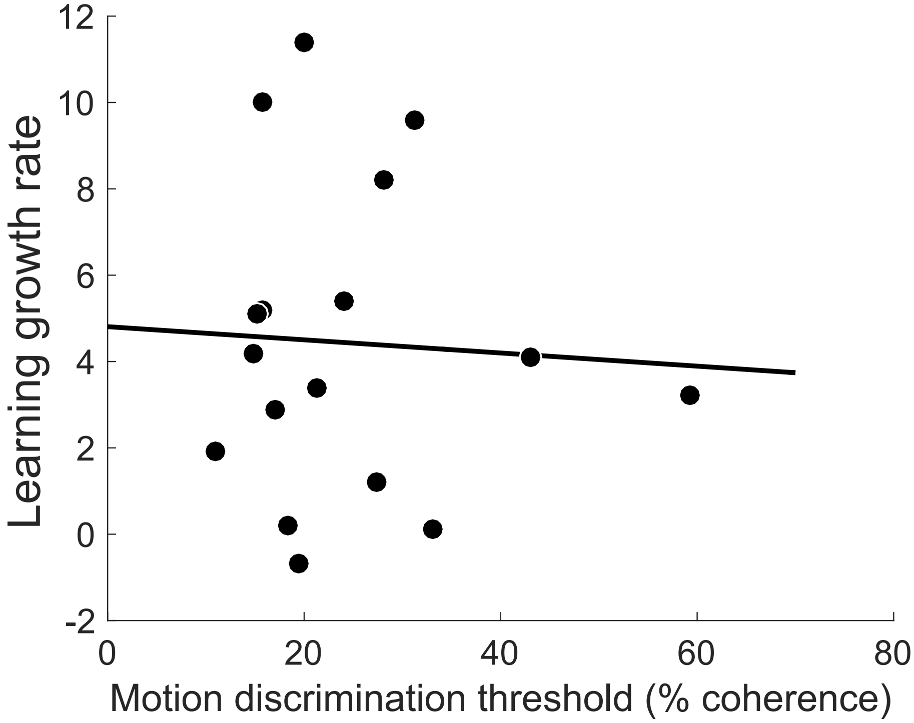

Supplementary Figure 3. Correlation between motion sensitivity and reading growth rate. Each individual's reading growth rate is plotted against motion direction discrimination threshold (percent coherence). The solid line represents the best-fitting regression line to the data. Motion sensitivity is does not predict improvements in reading skills over the intervention (r = 0.05, P = 0.84).
